# Key attributes of integrated community-based youth service hubs for mental health: a scoping review

**DOI:** 10.1186/s13033-019-0306-7

**Published:** 2019-07-23

**Authors:** Cara A. Settipani, Lisa D. Hawke, Kristin Cleverley, Gloria Chaim, Amy Cheung, Kamna Mehra, Maureen Rice, Peter Szatmari, Joanna Henderson

**Affiliations:** 1 Margaret and Wallace McCain Centre for Child, Youth and Family Mental Health, Centre for Addiction and Mental Health, Toronto, ON Canada; 2 Lawrence S. Bloomberg Faculty of Nursing, University of Toronto, Toronto, ON Canada; 3 Department of Psychiatry, University of Toronto, Toronto, ON Canada; 4 Hurvitz Brain Sciences Research Program, Sunnybrook Research Institute, Toronto, ON Canada; 5 McMaster University, Hamilton, ON Canada; 6 Department of Psychiatry, Hospital for Sick Children, Toronto, ON Canada

**Keywords:** Integrated care hubs, Mental health, Addiction, Youth, Young adults

## Abstract

**Background:**

Community-based, integrated youth service hubs have the potential to address some of the longstanding issues with mental health services for youth, including problems with access and system fragmentation. Better understanding of these approaches, particularly efforts to create a single point of entry to comprehensive, evidence-based services through youth service hubs, is needed to help guide future implementation and evaluation. This scoping review identifies the key principles and characteristics of these models of care, as well as the state of the literature, particularly with regard to implementation and replicability.

**Method:**

Electronic databases and grey literature sources were searched for material from 2001 to 2019, with diverse search terms capturing the concept of “integrated” or “one-stop shop” youth mental health services. Title/abstract and full text review were conducted, as well as additional focused searching. After screening 4891 texts at the title/abstract level and 496 at the full-text level, 110 documents were included for data extraction.

**Results:**

Several integrated care hub models for youth mental health services and related frameworks were identified internationally, largely in high-income countries. Common principles included an emphasis on rapid access to care and early intervention, youth and family engagement, youth-friendly settings and services, evidence-informed approaches, and partnerships and collaboration. Program characteristics also revealed similarities (e.g., providing evidence-informed or evidence-based services in youth-friendly spaces), with some differences (e.g., care coordination methods, types of service providers), potentially attributable to lack of available information about key ingredients. Outcome research was limited, with few rigorous evaluations of youth outcomes. Moreover, sufficient information for replication, community evaluation of feasibility or actual implementation was rarely provided.

**Conclusion:**

Internationally, integrated youth service hubs were found to share common key principles, while providing comprehensive services to youth with mental health difficulties. There is a great need for common language and measurement framework to facilitate replication, rigorous evaluation of outcomes, knowledge exchange, and dissemination of findings.

## Introduction

Mental health problems commonly start during childhood or adolescence, with about half of all lifetime cases starting by ages 14–15 and three-quarters by ages 18–24 [1, 2]. Worldwide, approximately 10–20% of children and adolescents experience mental health disorders [3, 4] and cumulative lifetime prevalence approaches 50% among adolescents and young adults in developed countries [1, 5, 6]. Mental health problems are also associated with high societal burden [7, 8]. Despite the clear need for mental health services, youth often do not receive evidence-based services in a timely, effective manner [9, 10]. Longstanding problems with youth mental health service provision, including system fragmentation, poor access to evidence-based services, discontinuation of support at the transition to adulthood and lack of an informed developmental perspective, could be effectively addressed by integrated care approaches offered across the adolescent and young adult developmental stages [11, 12]. Indeed, models of care that bring traditionally separate services together into one community-based setting to meet youth’s unique needs and span the transition to adulthood have recently gained the attention of researchers, service providers, and policymakers [13–15].

Integrated community-based youth service hubs (ICYSHs) reflect models of care that provide comprehensive, youth-focused services, including mental health services, health and other community and social services in a single community-based setting, sometimes referred to as “one-stop shops”, with “youth” defined to include both adolescents and young adults. Literature reviews of medical and behavioral health integration in primary care settings have been conducted [16, 17] and a meta-analysis suggests benefits [18]. However, although ICYSH models are being adopted internationally [13, 14], reviews of these integrated community-based models of care are lacking. One recent exception is a 2017 review by Hetrick et al. [19], which describes select programs and their outcome evaluation efforts. Authors highlight the need to further evaluate outcomes and develop best practice models [19]. They do not, however, examine the common principles across various exemplars of ICYSH. In addition, information on implementation of essential features of ICYSH models is needed to ensure that the components most crucial to their success are included in efforts to replicate the model and evaluate the feasibility and outcomes of the model in different contexts. This review identifies ICYSH models for mental health services, their characteristics, common principles, and aspects critical to implementation and replicability.

## Methods

### Search strategy and identification of relevant studies

**Fig. 1:**
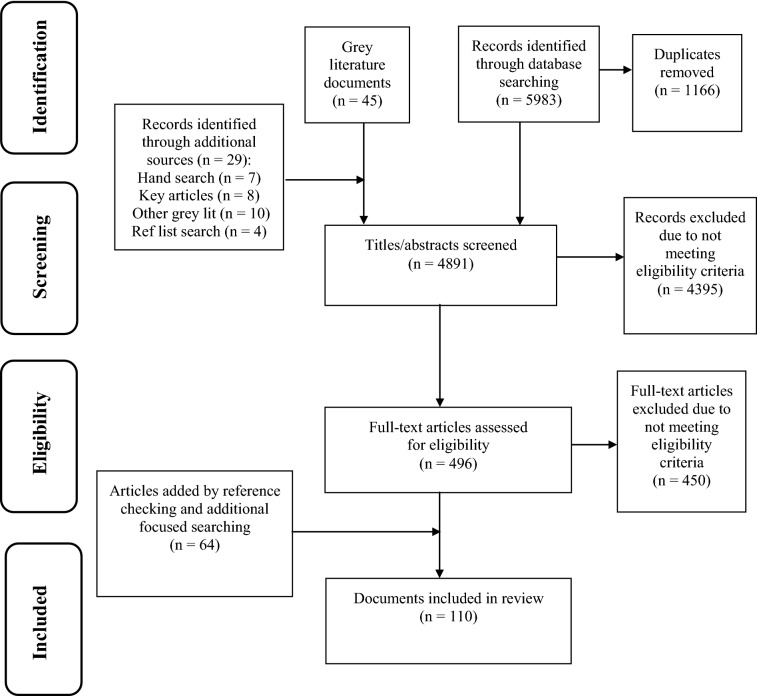
PRISMA flow diagram

Scoping review methodology was deemed particularly well-suited to examine the burgeoning area of ICYSHs. Scoping reviews comprehensively identify important concepts in a structured manner [20], including the extent, range, and nature of research activity in areas in which the available range of materials is unknown [21], e.g., when substantial grey literature is expected. The scoping methodology described in the literature was followed [21–23]. For a detailed description of the review methodology, see [24]. An electronic search was conducted in Medline, EMBASE, PsycINFO, CINAHL, ASSIA, and Campbell Collaboration Library and Cochrane Library using a combination of subject heading and text word terms for integration AND mental/behavioral health AND children/adolescents. Work published in English between 2001 and 2019 was included to capture recent developments in ICYSH models. Exclusion criteria included comments or notes, editorials, and letters. A total of 4817 unique citations were retrieved from the initial database search, after removing 1166 duplicates (see Fig. 1 for PRISMA diagram).

A grey literature search was undertaken following a comprehensive checklist [25]. A broad Google Advanced Search was performed and a focused search was conducted on specific youth-relevant mental health sites, resulting in 45 grey literature documents. An additional four documents were gathered by reviewing reference lists. The *International Journal of Integrated Care* was hand searched, yielding seven more articles. Eighteen additional works (eight articles and 10 grey literature documents) known to the authors were included, resulting in 4891 documents screened.

Inclusion criteria were as follows: presenting problem was mental or behavioral health, substance use concerns, or concurrent disorders; population was children, adolescents, youth, emerging adults, or young adults. The United Nations defines “youth” as the age range spanning from 15 to 24 [26]; the age range accepted for inclusion in the review was flexible to accommodate different definitions retained in the literature. Other inclusion criteria included models of care that brought together multiple service components to improve access to service, service quality, client satisfaction, efficiency [27], or youth outcomes [11], and aimed to make mental health systems or service delivery more fulsome [28]; services were coordinated, co-located (multiple types of services beyond mental health, all in a single setting), and community-based (not hospital, primary care, or school based). Non-research literature (e.g., policy documents) was also included to capture a broad range of work.

Titles and abstracts were independently screened by two raters, with project lead consultation to resolve discrepancies. Covidence [29], the Cochrane-recommended systematic review software, was used for title/abstract and full text review. Reasons for exclusion were: conference presentations presented > 3 years ago; adults or infants only (age < 2); not mental or behavioral health; not linked/integrated services; only hospital consultation-liaison services; only combination therapy-medication in a randomized control trial. Following title/abstract screening, 496 full-text articles were examined; of them, 450 documents were ineligible (see Fig. 1). At this stage, the authors checked references of included studies for other relevant work and conducted a focused search for related documents, adding 64 documents; 110 documents were ultimately included for data extraction. Information was extracted from these documents using a data extraction tool which was iteratively modified based on the knowledge gathered during the scoping review process (see published scoping review protocol for more information [24]). Members of the research team repeatedly read the extracted data, returning to the full text for context, in order to synthesize the findings by consensus. Following extraction, data were reviewed to identify unique programs and examine the extent of information available for each program.

## Results

In total, 87 journal articles and 23 grey literature documents were included in the review. The primary purpose of journal articles was: description of ICYSH model, 30; model research, 42; implementation descriptions, 9; RCT protocol, 1; position papers, 4; review, 1. The diverse grey literature included: government-commissioned reports, reviews and service frameworks, 5; information related to a program model, efforts, and progress, 6; informal program information, 4; in-depth program descriptions/reports, 3; independent program evaluations, 4; model recommendations, 1. In all, 94 documents described components of 8 unique programs (Table 2). Insufficient information to meaningfully review was provided about 11 models, resulting in their exclusion from the detailed analysis in this report, although they are described in summary Table 1.

**Table 1:** Overview of all integrated community-based youth service hub (ICYSH) models meeting inclusion criteria in the review

Program	Location	Year established	General program summary
ACCESS Open Minds [13, 19, 38–41, 97]	Canada (national level)	2014	Applies evidence to bridge science-practice divide and meet goals of transforming mental health care and producing better outcomes Considers contextually driven circumstances to address deficits in youth mental health services and uses culturally appropriate practices Aims to provide early case identification, rapid access to initial assessment, continuous service bridging adolescence and young adulthood, and connection to specialized services
Child Health Centre [66]	Israel	1984	Provides comprehensive, integrated health care services at the community level Consults on school problems, behavioral concerns, and peer and family relationships
Corner Clinic Teen Parent Programme [87]	Ypsilanti, Michigan, United States	2008	Serves teenage mothers’ medical, social, and psychological needs through multimodal, collaborative program Involves individualized care and group support Provides developmental screening for children
Forward Thinking Birmingham [13, 14, 37]	Birmingham, UK	2015; 2011 (piloted as Youthspace)	Applies principles of prevention, choice, and personalized care Provides a dedicated youth mental health service Engages young people through rapid response and high-quality initial assessments
Foundry [19, 42, 43, 75, 76, 79, 97, 98]	British Columbia, Canada	2015	Ensures health promotion, prevention and early intervention are core components of a comprehensive system of care Strives to provide services that are timely, accessible, developmentally appropriate, socially inclusive and equitable, and culturally sensitive as well as youth- and family-centered, collaborative, and empowering Allows for service integration through partnerships and collaborative inter-sectoral working and focuses on integration process Strives to provide services that are evidence- and trauma-informed and effective
headspace [12–14, 19, 29–32, 45–48, 50, 51, 52–56, 59, 60, 64, 69, 70, 73, 74, 80, 81, 84, 90, 90–94, 97–118]	Australia (national level)	2006	Strives to meet core health needs through highly accessible, multidisciplinary model of care Bridges gap between mental health and substance services through co-location and common governance Provides early intervention within enhanced primary care structure/one-stop shop linked to specialist services, schools, and other community-based organizations
Isle of Wight service [68]	Isle of Wight, UK	2004	Aims to meet the needs of children at risk for requiring residential services in the community Co-locates four agencies (health, education, social services, substance misuse) into one service
Junction [119]	UK	2004	One of the eight participants in the Youth Crisis project Provides mental health services for 16–25 year olds Provides easy-to-access, swift response with low wait times for youth in crisis situations
Jigsaw [13, 14, 19, 33–36, 45, 49, 57, 120]	Ireland (national level)	2008	Integrates supports and services for young people through community capacity building Engages young people in design and planning of integrated services Improves availability of programs that teach young people core competencies and resilience, and strives to identify those at risk earlier Ensures clearly defined pathways to care and engages community leaders
Northern Ireland Care Trusts [121]	Northern Ireland	2002	Provides a single point of entry for mental health referrals, improves referral and assessment process, and reduces wait times and service duplication through fully integrated, comprehensive health and social care trusts formed by integration of existing provider trusts Serves health and social care needs through one-stop shop, community-based well-being and treatment centers
Oak House Child Development Centre [67]	Isle of Wight, UK	2001	Provides range of support for children with complex difficulties through interagency working between health, education, and social care Expands initial focus on children with autism spectrum disorders to include wider range of difficulties Provides coordinated approach to assessment and diagnosis, support and intervention planning, and service delivery
Orygen Youth Health [12, 14, 33, 61, 63, 72, 78, 83, 90, 120–124]	Melbourne, Australia	2002	Provides early intervention for psychosis, mood disorders, and borderline personality disorder through evolution of Early Psychosis Prevention and Intervention Centre (EPPIC) model Provides triage, assessment, and crisis response 24/7, and community and home-based services through a youth access team Delivers early intervention services over a 2 year period of care through four specialized clinics
Plan d’action en santé mentale (Mental Health Action Plan) [71, 125]	Quebec, Canada	2005	Emphasizes primary care as entry point to mental health care and avenue for mental health service delivery Supports primary care providers through collaborative care or shared care model involving partnership between first-line health and social service care providers and specialized mental health resources
Spilstead Model [85]	Sydney, Australia	2005 (study period commenced)	Provides holistic and intensive child- and parent-focused services and interventions within one-stop shop Targets families with complex parental issues and children under school age experiencing social, emotional, or developmental delays
YouthCan IMPACT [19, 44, 52, 88, 97]	Toronto, Canada	2016	Provides range of youth-friendly services in one setting utilizing rapid, stepped-care approach Delivers personalized care for youth with mental health and substance use concerns in their community Seeks to address service gaps, decrease wait times, be more youth and family friendly, and be more cost-effective Evaluates effectiveness of model through pragmatic randomized controlled trial
Youth One Stop Shops [19, 38, 62, 65, 82, 126, 127]	New Zealand	1994	Provides accessible, youth-friendly health, social and other services in a holistic ‘wraparound’ manner at little or no cost, in a safe and welcoming environment Wraps range of services around youth to meet individual needs in a seamless and coordinated way Delivers strengths-based services in a manner that is non-judgmental, culturally appropriate, and respectful to youth utilizing youth developmental principles
Youth Stop (YStop) outreach clinic [128]	City of Greater Dandenong, Melbourne, Australia	2010	Provides early intervention and intake not dictated by diagnosis within youth outreach ICYSH inspired by headspace ideals Addresses existing gaps in youth mental health delivery by linking primary care services and the tertiary level mental health program
Youth Wellness Centre [86]	Hamilton, Ontario, Canada	2015	Emphasizes accessibility, peer support, family support, use of technology, youth participation, evidence-based treatment, efficiency, and system linkages within early intervention, youth-focused service Includes service streams for early intervention, transition support, mobile team, and re-engagement Co-locates service with substance use counseling and monthly primary care clinic
Youth Wellness Hubs Ontario [97, 129]	Ontario, Canada	2017	10 sites providing integrated, stepped-care model with mental health, addiction, primary care, community and social services for youth 12–25 years of age Provides rapid access, evidence-based services that are co-created with youth, caregivers and service providers

*ICYSH* Integrated Community-based Youth Service Hub, *UK* United Kingdom, *EPPIC* Early Psychosis Prevention and Intervention Centre

### Summary of models reviewed

Multiple international ICYSH models were identified (Table 1). Although literature was not restricted by country of origin, the documents included in the review were found to be based on ICYSH from high-income countries. Programs include: headspace and Orygen Youth Health in Australia, Jigsaw in Ireland, Forward Thinking Birmingham (formerly Youthspace) in the United Kingdom, Youth One Stop Shops in New Zealand, and YouthCan IMPACT, Foundry, and ACCESS Open Minds in Canada. See Table 1 for information on all models identified.

The Australian headspace model aims to establish a multidisciplinary, primary mental health services model that is highly accessible and specialized to meet the core needs of young people [30, 31]. It also aims to bridge mental health service gaps through co-location and common clinical governance. A key principle is that youth are not turned away from services based on severity or diagnosis. headspace seeks to provide early intervention within an enhanced primary care one-stop shop that is closely linked to local specialist services, schools, and other community-based organizations [14]. As of early 2019, there are a total of 110 headspace centers established in Australia [32].

Orygen Youth Health in Australia evolved out of the Early Psychosis Prevention and Intervention Centre (EPPIC) model [14]. This service seeks to bridge the gap between child/adolescent and adult mental health services in the Melbourne area [33]. Four specialized clinics focus on youth with first episode psychosis, mood disorders, emerging borderline personality disorder, and youth at ultra-high risk for a psychotic disorder [14]. Each clinic provides a full complement of interventions over a 2-year period of care. A youth access team provides triage, assessment, and crisis response 24 h a day, 7 days a week, as well as community and home-based services [14].

Headstrong, the National Centre for Youth Mental Health in Ireland, established Jigsaw [34] to integrate supports and services for young people through community capacity building, with 13 centers currently providing evidence-based services [35]. Key efforts include engaging young people in service design and planning to improve responsivity to youth needs and minimize stigma, building an integrated community network, improving the availability of programs that teach resilience and core competencies (e.g., interpersonal skills), identifying those at risk for mental health problems earlier, ensuring clearly defined pathways to care, and engaging community leaders [34, 36].

In England, Forward Thinking Birmingham advocates prevention, choice, and personalized care and offers youth a range of new services and facilities; this model will likely serve as a United Kingdom template for additional youth mental health service reform efforts [13]. Forward Thinking Birmingham is based on the success of pilot initiative Youthspace, which explored effective clinical approaches among youth up to age 25 [37]. Goals included providing rapidly responsive, youth-friendly early intervention services and utilizing a range of treatments.

New Zealand’s Youth One Stop Shops were developed independently in response to local needs and opportunities supported by relationships with other providers and funders, though they share a common goal of promoting youth access to healthcare and social services [38]. In 2009, there were at least 14 Youth One Stop Shops across the country, providing a range of youth-friendly, holistic services at little or no cost to the youth.

Several ICYSH efforts are underway in Canada. ACCESS Open Minds is implementing 14 ICYSHs in urban and rural areas across Canada [13, 39, 40]. The multi-stakeholder ACCESS network includes youth, families, community organizations, service providers, researchers, and policy makers, and provides rapid access to evidence-informed, youth-friendly services appropriate for varying levels of need [40, 41]. Foundry is a British Columbia model that co-locates health and social services through a network of centers and e-health services [42]. By supporting communities to develop youth-friendly ICYSH storefronts, working with provincial resources, and linking to specialized services and community agencies, Foundry aims to strengthen a comprehensive system of care for youth and young adults provincially and improve access to mental health, substance use, and primary care services [43]. YouthCan IMPACT in Toronto involves the development, implementation and evaluation of an Integrated Collaborative Care Team (ICCT) model consisting of linked components that are co-located within community-based, youth-friendly ICYSHs [44].

### Model characteristics

#### Populations

See Table 2 for a summary of the characteristics of ICYSH models. Many programs focused on adolescence through early adulthood. For example, headspace and Jigsaw include youth ages 12–25 [30, 31, 36, 45], and ACCESS Open Minds serves youth ages 11–25 [38–41]. The 15–17 age range is the peak age of presentation at headspace [45–48] and Jigsaw [49], with an overrepresentation of females (e.g., headspace, 63.7%) [45–48]. However, nearly half of youth at Jigsaw were male (43.5%) [49]; there have been other encouraging findings regarding male youth accessing ICYSHs, despite evidence of greater difficulty engaging males in traditional mental health (MH) treatment [34, 50]. Service access at headspace is improved for youth who are socially and economically marginalized as compared to the general population, or those youth who lack support from their families and peers; however, young adults (18–25) and those from non-English speaking households are underrepresented [50].

**Table 2:** Characteristics of integrated community-based youth service hub models of primary focus in the review

Program	Population	Setting characteristics	Service providers	Services and interventions	Infrastructure and care coordination methods
ACCESS Open Minds (CAN) [13, 38–41]	11–25 years Youth with established or emerging mental health problems Any type of mental health problem, mild to severe	Youth-friendly physical spaces serve as portals for help-seeking and venues for peer support activities	ACCESS-trained clinicians (healthcare professionals who are not physicians) Youth and family peer support workers	Evidence-informed interventions staged by phase of illness and level of care needed Ranges from minimal support and basic psychosocial interventions to care for common and severe disorders, through connections to specialized services Tele-monitoring, remote specialist consultations, and electronic specialized interventions as needed	ACCESS clinician connects youth to appropriate services by referring to further specialized care if needed, introducing youth to specialists and, accompanying them and their families to initial appointments as needed
Forward Thinking Birmingham (UK) [13, 14, 37]	0–25 years Range of common mental health conditions	Non-stigmatizing, youth-friendly environment	Consultant psychiatrist Not further specified in documents reviewed	Youthspace included assessment and diagnostic formation followed by brief CBT and symptomatic treatment via medication by GP, with consultation team advising Community partnerships provided activities to reduce NEET status Access to on-line support and information, specialized intensive care streams when needed	Not specified in documents reviewed
Foundry (CAN) [42, 43, 75, 76, 79, 98]	12–24 years Mild to moderate mental health and substance use problems	Non-traditional settings (e.g., shopping centers, store fronts)	General practitioners, nurses and nurse practitioners, psychiatrists, social workers Peer support workers	Primary care, sexual health, mental health, substance use counseling Evidence-based practices Psychosocial rehabilitation, housing support, income assistance, and peer support through partnerships	Collective impact approach, involving centralized infrastructure and structured processes to coordinate ongoing collaboration, (e.g., data capture systems for cross-partnership service integration, clinical care, research, evaluation) Tele-health and information sharing guidelines to increase effectiveness and coordination of team-based care
headspace (AUS) [12–14, 29–32, 45–48, 50, 51, 52–56, 57–60, 64, 69, 70, 73, 74, 80, 81, 84, 90, 90–94, 97–118]	12–25 years Not diagnosis specific Majority present with high or very high levels of psychological distress 19–29% of young adults with NEET status	Centrally located, close to public transport Youth-friendly, relaxed environments: couches and bean bag chairs, colorful walls, creative artwork Less white space characteristic of traditional health care settings, open waiting area, open center spaces, and high ceilings Recreational activities	General practitioners, psychologists, counsellors at all centers Other specialist practitioners, such as psychiatrists or sexual health workers at some centers Youth workers or social workers at some centers Mental health nurses, occupational therapists, vocational support workers, and Aboriginal health workers	Four core service streams of mental health, drug and alcohol services, primary care, and vocational assistance CBT most common treatment provided for all presenting concerns, followed by supportive counseling (not including youth with features of borderline personality disorder), and psychoeducation Brief Intervention Clinic provided at some centers Enhanced headspace services provide evidence-based early intervention services for psychosis (headspace Youth Early Psychosis Programs)	All youth enter data electronically before each service occasion (Minimum Data Set process) headspace national office guides infrastructure efforts (e.g., funding and assessment guidelines, contracts, reporting structure and performance indicator development, policies, partnership documentation, memorandums of understanding, governance guidelines, business model guide) Youth Access Clinician screens youth, becomes coordinating clinician, and provides brief interventions and supports
Jigsaw (IRL) [13, 14, 33–36, 45, 49, 57, 120]	12–25 years, most common age: 16 years Not diagnosis specific High levels of psychological distress Many 21–25 year olds unemployed More females than males in brief interventions Majority of youth attend school, live with families, have married parents	Youth café facility provides a public space for a youth, a setting for program delivery, and a pathway to mental health and health supports	Variety of allied health professionals, such as psychologists, OTs, social workers, and mental health nurses Psychiatrists, GPs, youth workers, family therapists, drug counsellors at some sites Wraparound facilitators already engaged with youth in various contexts	Individual case consultations (indirect support) and brief contacts Brief interventions (1–6 sessions), CBT-informed and solution-focused Extensive engagement processes commonly addressing emotional, cognitive and behavioral self-regulation, substance abuse, learning and family issues Best-practice and evidence-based interventions Peer support Prevention programs Social, recreational, and work related programs	Youth data captured through online system designed to record important clinical, case management, service delivery, and outcome-related information Wraparound facilitator to ensure youth accesses necessary services Psychiatrists or nurses link youth with services at primary care or community mental health clinic if needed Outreach and support workers maintain connection and follow up with youth even after they engage with other services
Orygen Youth Health (AUS) [12, 14, 33, 61, 63, 72, 78, 83, 90, 120–124]	15–25 years Psychosis, mood disorders, borderline personality disorder	Drop-in peer support and resource room designed and decorated by youth, co-located with outpatient services IMYOS provides services in most natural setting, often home, school, public locations	Case manager is main point of contact and is a mental health nurse, clinical or provisional psychologist, OT, or social worker Psychiatrists or psychiatric registrars. Peer support workers Hospital chaplains	Four specialized clinics offer 2 years of care and full range of interventions, including case management, individual support and therapy, and consultation–liaison; work closely with psychosocial recovery program Embedded forensic consultation pilot program to better manage and reduce risks of violence Crisis intervention and home-based treatment when needed Additional inpatient service focused on acute care and brief admissions	Single, shared health record Case manager links youth to services within and outside Orygen Youth Health Duty workers support with urgent matters if case worker unavailable
YouthCan IMPACT (CAN) [44, 52, 88]	14–18 years (study age range) Not diagnosis specific Youth with MH challenges	Community mental health sites Youth friendly spaces	Youth workers, social workers, care navigators, peer support workers, primary care providers High intensity psychiatric response: psychiatrists, NPs	Solution-focused brief therapy (SFBT) and group DBT for youth and family members Primary care, high intensity psychiatric response Care navigation, assertive outreach Peer support, e-health support tools, 24/7 crisis text support	Care navigator works with specialists to coordinate care, ensure continuity, and support transitions between systems such as education and justice, adolescent and adult mental health
Youth One Stop Shops (NZL) [38, 62, 65, 82, 126, 127]	10–25 years Not diagnosis specific	Centrally located Non-specific signage to reduce stigma	Doctors, nurses Youth workers and mentors, peer support workers Counselors, social workers, psychologists	Primary care, sexual and reproductive health, mental health, drug and alcohol services, counselling, smoking cessation, family planning, health promotion and education services Social services, including vocational, education, and training assistance, housing support Legal services, parenting and youth transition services CBT and motivational work	Youth workers facilitate access to all services, providing a bridge between the youth and needed services, and assist in coordinating care to optimize outcomes Early electronic flagging of clients as they turn 24 years to prompt transition planning

*CBT* cognitive-behavioral therapy, *DBT* dialectical behavioral therapy, *NEET* not in employment, education or training, *IMYOS* Intensive Mobile Youth Outreach Service, *SFBT* solution-focused brief therapy, *GP* general practitioner, *OT* occupational therapist, *NP* nurse practitioner, *CAN* Canada, *UK* United Kingdom, *AUS* Australia, *IRL* Ireland, *NZL* New Zealand

Most models identified a focus on intervening during early stages of distress, possibly before diagnostic criteria are met [13, 36, 39, 40, 43, 51, 52]. In headspace centers, mood and anxiety symptoms and disorders were the most common presenting problem [46, 52–55], although formal diagnosis was available for only few clients [46]. Additionally, 69–73% of headspace youth presented with high or very high levels of psychological distress [46, 48], and 19–29% of young adult clients were not engaged in education, employment or training [46, 56]. Jigsaw similarly reported seeing youth with high levels of psychological distress [49], with four main types of issues identified among youth: developmental, comorbid, anxious and externalizing disorders [57].

#### Settings

Documents varied in the level of detail provided about service settings. Several documents emphasized efforts to make settings accessible to youth, non-stigmatizing, and youth-friendly. For example, some programs were located in shopping centers and storefronts and involved youth in decisions about design and décor [43, 58]. Others were centrally located or close to public transportation [31, 38, 50]. In headspace centers, physical aspects of the settings described as youth-friendly include couches and bean bag chairs, colorful walls and creative artwork for a vibrant look, and an open waiting area and high ceilings to promote a feeling of safety and openness [59]. Youth-friendly environments were key for Foundry, including an informal, non-clinical space where youth can spend time with peers in addition to attending appointments [43]. Jigsaw discussed plans to develop a youth café with a public youth space and a program delivery area [34]. Some headspace centers encourage recreational use with pool and air-hockey tables [60].

#### Service providers

Documents describing the types of service providers typically include a range of professionals from several disciplines. For example, the intensive mobile youth outreach service subprogram of Orygen Youth Health consists of two psychologists, two social workers, one occupational therapist, one psychiatric nurse, and a part-time consultant psychiatrist [61]. Very few documents provided detailed information about service providers’ roles. Within New Zealand’s Youth One Stop Shops, several services are offered by physicians, nurses, counsellors, social workers and youth staff [62], providing primary care, sexual and reproductive health, mental health, drug and alcohol services, counselling, smoking cessation, family planning, and health promotion and education services [38]. For the Foundry prototype, primary care and sexual health services are provided by general practitioners and nurse practitioners, mental health services by psychiatrists, and substance use counseling by social workers and nurses. Additionally, partnerships provide other services, including psychosocial rehabilitation, housing support, income assistance, and peer support. ACCESS Open Minds clinicians include professionals with psychology, nursing, social work, or occupational therapy backgrounds who assess youth needs and provide need-based care [40, 43]. In YouthCan IMPACT, each integrated collaborative care team includes youth workers, social workers, psychiatrists, nurse practitioners, peer support workers, access to primary care providers, and a care navigator [52]. For higher-risk youth, psychiatrists and/or nurse practitioners provide psychiatric assessment and medication management, along with other clinically appropriate interventions. Several ICYSH models in this review included peer support workers as service providers [38, 40, 43, 52, 63], reflecting the importance of youth with lived experience supporting other youth experiencing similar issues.

Many documents did not provide information on their service provider training process. Some documents broadly indicated that there is training in evidence-based interventions and best practices [36, 59, 64], in working with youth and youth-friendly approaches [59, 65], and professional development or continuing education activities [38, 66, 67], though difficulties with releasing staff for professional development opportunities were also noted [38]. Some documents mentioned ongoing supervision and regular team meetings [51, 68]. As part of Jigsaw, a structured training program addresses adolescent development and mental health, strengths-based and solution-focused approaches, risk assessment, goal setting, crisis response, interagency working and collaboration, consultation processes, and integrated planning and evaluation [34]. Initial training ensures a shared understanding of the Jigsaw philosophy and implementation process, with the plan of extending the training more broadly to others in the community who work with youth to increase service capacity.

#### Services and interventions

More information was provided on broad types of services included in ICYSHs than on specific interventions (Table 2). headspace includes four core service streams: mental health, drug and alcohol services, primary care, and vocational assistance (e.g., [31, 69, 70]). Primary care is a component of several other models (e.g., [38, 43, 71]). Social services, including vocational assistance, education and training support, and housing support were a component of other models [37, 38]. Several models use brief, solution-focused interventions (e.g., [13, 34, 37, 45, 52]), as well as peer support and mentoring (e.g., [34, 52, 72]).

Some models provided information on specific psychosocial interventions utilized. Services provided by Jigsaw include case management, problem-solving, mindfulness, cognitive-behavioral therapy (CBT), substance use services, psychoeducation, and social skills [57]. In headspace centers, CBT was reported to be the most common treatment provided for all presenting concerns, followed by supportive counseling and psychoeducation [73]. In YouthCan IMPACT, standardized protocols are used for interventions including solution-focused brief therapy (SFBT) and group dialectical behavioral therapy (DBT) skills [52]. Early intervention approaches for psychosis including CBT were mentioned as part of enhanced headspace services, ACCESS Open Minds, and Orygen Youth Health [13, 14, 41]. ACCESS Open Minds provides services depending on a youth’s needs, including specific therapies (e.g. DBT for emotion dysregulation), transition to a specialist service (e.g. eating disorder program), or admission to a hospital (e.g. high risk of suicide) [40]. Some documents mentioned the use of evidence-based approaches, but not specific interventions (e.g., [42, 49]). For example, an independent headspace evaluation interim report indicated that implementation efforts were prioritized over tracking the extent of use of evidence-based treatments [74].

Intake and assessment processes were described in some papers; however, little information was provided on the use of standardized assessment measures. One exception, however, is the use of Kessler Psychological Distress Scale (K-10), Clinical Global Impression (CGI) scale, and the Social and Occupational Functioning Assessment Scale (SOFAS) by ACCESS Open Minds clinicians as part of a measurement-based care approach [40]. Some models mentioned following a stepped-care or clinical staging model (e.g., [51, 52]). Other work did not use this terminology, but described analogous processes. For example, headspace interventions are said to be delivered according to severity and complexity of an individual’s needs [64]. Some documents discussed personalized treatment plans [37] and person-centered approaches [42].

#### Infrastructure and care coordination

Infrastructure aspects included use of health registries, outcome tracking, information sharing through electronic records, and cross-organization administrative processes, although many documents did not discuss infrastructure.

The Foundry model uses a centralized infrastructure and structured processes that coordinate ongoing collaboration, such as data capture systems that support cross-partnership service integration, clinical care, research, and evaluation [43]. Tele-health and information sharing guidelines are promoted to increase the effectiveness and coordination of team-based care [75, 76]. At headspace, data are collected through a minimum dataset process in which all youth accessing centers and their service providers enter data electronically at each service occasion [31, 46]. The headspace national office is responsible for infrastructure efforts including developing funding and assessment guidelines, negotiating contracts, developing reporting structures and key performance indicators, and providing policies and tools including partnership documentation, memorandums of understanding, governance guidelines and a business model guide [74]. In Jigsaw, youth data are captured through an online system that records clinical, case management, service delivery, and outcome-related information [49]. The use of a single, shared health record to facilitate collaboration and streamline care was mentioned in several documents (e.g., [58, 66, 72]). Shared infrastructure has been identified as positively contributing to service coordination and integration [74].

Other methods of care coordination include identifying point persons to facilitate service access and coordinate care across specialists, with titles such as care navigators, youth workers, and youth access clinicians [38, 51, 52, 77]. These workers engage in activities such as exchanging information and participating in joint coordination meetings and planning activities [74]. Some models discussed the role of support or outreach workers in linking clients to services outside the ICYSH and maintaining connection and follow up [34, 72]. Coordinating external referral pathways was viewed as helpful by youth accessing headspace services [59] and care coordination is a key headspace performance indicator [70].

### Common principles of integrated care models

Several common principles of ICYSH models were discussed, including improving access to care and early intervention, youth and family engagement and participation, youth-friendly settings and services, evidence-informed approaches, and partnerships and collaboration (Table 3).

**Table 3:** Application of common principles of integrated community-based youth service hub models of primary focus in the review

Program	Improving access to care and early intervention	Youth and family participation and engagement	Youth-friendly settings and services	Evidence-informed approaches	Partnerships and collaborations
ACCESS Open Minds	Provides timely access, early identification [39, 40] Employs clinician as single contact point for direct, rapid access to assessment (72 h; [40, 41]) Utilizes telemedicine for rapid remote assessment [40, 41] Requires no referral or specific symptom [40, 41]	Engages youth and families in project conceptualization, core values, implementation, and research [39, 40] Includes youth as full care partners and encourages participation of youth and families at all levels (e.g., peer support, service planning, evaluation, research [40, 41])	Commits to a youth-friendly, empowering culture [40, 41] Offers youth-friendly physical spaces called *Youth Space* as portals for help-seeking and venues for peer support activities [13, 40]	Provides access to evidence-informed mental health care [39] Utilizes needs-based staging model [40, 41] Generates evidence and tests effectiveness of service model [13, 40]	Forms coalition of partners integrating research into care [39, 40] Includes network of youth, family/carer, community, service provider, researcher, and policy/decision maker stakeholder groups [40, 41] Utilizes partnerships with emergency/hospital services [40, 41]
Forward Thinking Birmingham	Provides rapid response and assessment/diagnostic formation to GP within 1 week [13, 37] Improves access to effective support and provides range of services based on principles of prevention/early intervention [13]	Consults extensively with youth and conducted qualitative research on experiences with existing services [14] Designs website for advice, education, and individualized assessment based on youth input [14]	Emphasizes non-stigmatizing, youth-friendly environments and services [37]	Uses CBT as default evidence-based intervention [14]	Leverages partnership with agency focused on education, skill training, entrepreneurship, social inclusion and employment [14] Creates consortium of NHS partners (child/adult services), voluntary sector, and private healthcare organization [13]
Foundry	Improves access to youth mental health, substance use, and primary care services [43] Offers drop-in services [43] Provides online access and local walk-in centers [42] Improves service providers’ and community members’ knowledge of how to access services [76] Includes prevention and early intervention as core service components [43]	Designs service delivery and makes decisions with youth participation to reduce service barriers, better meet needs, and promote youth-friendly approaches [43] Involves youth in staff recruitment; utilizes Youth Advisory Groups [43, 98] Promotes engagement activities (e.g., focus groups and journey mapping) to make youth and families’ experience central to the process [75, 79]	Utilizes youth-friendly storefronts, non-stigmatizing centers; offers accessible hours and preferable locations [43] Employs friendly health and service providers [42]	Utilizes evidence-based best practices [42] Uses integrated e-health based on emerging evidence [43] Links expanded implementation to outcomes and evaluation results [43]	Forms governing council based on partnership of several organizations [43] Utilizes partnerships for peer and housing support, income assistance, phone/chat/email/text supports, and online therapies [43] Plans collaborations for public health approaches and Aboriginal youth needs [43] Partners with school districts and communities for mental health awareness [75]
headspace	Offers highly accessible model of care [30, 31] Provides physical health services to allow for stigma-free access point [14] Utilizes clinical staging approach to support early and pre-emptive intervention [90]	Develops national and local youth participation initiatives [31, 59] Consults youth advisory groups about various matters (e.g., youth friendly environment, staff recruitment, operational decisions; [31, 59, 74]) Solicits feedback from youth and families through exit surveys, audits, websites and social media, feedback box, and focus groups [31, 70, 80] Promotes youth decision making about care and peer support [31, 106] Assesses satisfaction and identifies barriers [80, 110]	Prioritizes youth participation to achieve youth friendliness [74] Creates youth-friendly atmosphere: youth art, structural building changes, youth-friendly location, information of interest to youth in waiting area [31, 70] Facilitates youth participation in mental health care via physical set-up of clinics and being made to feel welcome by all staff [31, 60]	Provides evidence-based care within a clinical staging framework [31, 90] Utilizes CBT-based interventions for depression, anxiety, and psychosis, and motivational interviewing and behavior contingencies for substance use [90] Provides CBT most often for all primary concerns [73] Plans to produce evidence-based resources to support sites in using evidence-based practices [90]	Designs and develops model with input from consortium of research and academic institutes, practice network, and psychological society [90] Links to local specialist services, schools, and other community-based organizations [31, 90] Identifies key agency to lead each center on behalf of local consortium of organizations who coordinate and deliver four core service streams [14]
Jigsaw	Offers diversity of access pathways, often self- and parent-referral [14, 57] Provides central location and immediate response [34] Offers accessible prevention and early intervention services [34, 36]	Engages youth in design, implementation, and review of programs and conducted extensive youth consultation process [34, 57] Involves Youth Advisory Panel at each site and Youth Participation Officer in design and planning process of each initiative [34] Engages youth to increase likelihood services are relevant, stigma-free, and accessible [36]	Sees youth in their usual settings; plans youth café facility [34] Consults youth on creating welcoming and approachable sites (e.g., colour schemes, language to describe program, process for entering site, balance between professional and relevant for youth; [36]) Offers youth-friendly, storefront drop-in center [120]	Supports service providers in providing evidence-based, best practice approaches [36, 57] Exposes sites to youth mental health literature to promote adoption of best practices and reviewed evidence-based programs and research literature in development process [36]	Fosters partnerships among services [36] Engages and partners with all relevant stakeholders, including key statutory, community, and volunteer agencies, and establishes new partnerships [14] Develops positive partnerships with government [34]
Orygen Youth Health	Matches target age with peak MH onset [12] Provides early intervention for psychosis, mood disorders, and borderline personality disorder [14] Offers 24/7 triage, assessment, and crisis response [14]	Facilitates regular meetings of current and past clients to improve service, produce newsletter, and participate in selecting staff [63, 122] Offers mobile outreach services (IMYOS) for high-risk youth with complex needs and history of poor engagement with clinic-based services [61, 78, 83] Provides flexible interventions and engages family [124]; designs interventions with youth and families’ guidance [61]	Advocates youth-friendly approaches within IMYOS [61] Fosters youth-friendly culture [14] Provides peer support drop-in room [63, 122]	Provides evidence-based mental health care [14] Focuses on development and delivery of best- practice interventions within IMYOS, though overall approach without documented evidence base [61] Uses cognitive therapy routinely [61]	Links with other mental health and general support agencies essential to ensure quality service delivery [14] Provides pilot forensic services through consulting clinicians from specialist institute [123]
YouthCan IMPACT	Offers MHA services within walk-in clinic/one-stop shop [44] Improves access to timely services through community-based stepped-care model [44] Provides an early, low intensity intervention through solution-focused brief therapy [52]	Develops model with key participation of youth and family members with lived experience of the mental health system [52] Engages youth in all aspects of the project, such as designing the research study, (i.e., advising how to measure improvement) and choosing clinical services offered [88]	Provides services in youth-friendly environments in the community [52]	Links several evidence-informed components within model [52] Includes evidence-informed interventions, namely, solution-focused brief therapy, youth- and family-focused DBT, and peer support [52]	Develops model in partnership with several community agencies, adolescent psychiatry departments, academic collaborators, and other stakeholders [52] Partners with outreach services and targeted intervention programs [52] Encourages partners to join a common culture and respects unique strengths [44]
Youth One Stop Shops	Promotes access to range of health care and social services [38] Improves access through outreach, mobile, satellite services, and/or evening hours; provides transport at times [38] Offers services in central locations, close to public transport and other places youth frequent [38] Utilizes youth workers to serve as a communication bridge [38]	Opens site with youth-driven efforts: commissioning needs analysis, forming a trust and lobbying for funding, and employing staff [126] Facilitates youth focus groups; involves youth in activities such as designing a youth health information card [65] Involves youth at all levels (e.g., service evaluation, decision-making processes [38])	Offers youth-friendly opening hours to accommodate school and work [38] Provides facilities attractive to youth (e.g., couches, music, recreational activities; [38]) Employs staff skilled in interacting with youth, responding to needs [38, 127]	Utilizes CBT [65]	Links with several organizations formally and informally within and outside the health and disability sector; allows for varying types of relationships and information exchange [38, 127] Enables through linkages co-location of services, collaboration on community projects and events, development of resources, and cross-trainings [38] Facilitates better service transitions through collaborations with mental health services [126]

*GP* general practitioner, *CBT* cognitive-behavioral therapy, *DBT* dialectical behavioral therapy, *IMYOS* Intensive Mobile Youth Outreach Service, *NHS* National health Services, *MH* mental health, *MHA* mental health and addiction

#### Improving access to care and early intervention

A number of programs emphasized timely access to care, with the ACCESS Open Minds providing access to assessment within 72 h and appropriate care within 30 days [40, 41], the Forward Thinking Birmingham Program providing rapid response and assessment within 1 week [13, 37], and the Orygen Youth Health program providing a 24/7 triage, assessment and crisis response [14]. In addition, diverse access pathways were provided, e.g., self-referral [14, 31], walk-in or drop-in services [41–44], and online access to services [48] including telemedicine [41, 42]. Similarly, a number of programs highlighted the importance of early intervention [14, 31, 34, 36, 39, 43, 52, 78] and some included clinical staging [78] or stepped-care [52] to provide services at the earliest stage necessary.

#### Youth and family engagement

Multiple programs mentioned involving youth and families in design, implementation and evaluation of ICYSH models [14, 31, 34, 37–40, 43, 52, 57, 61, 74, 79, 80]. The programs established Youth Advisory Groups [31, 34, 43, 59, 74] or conducted focus groups [65, 70, 75] and surveys [70], and collected feedback from social media/websites [70] to ensure youth and family engagement.

#### Youth-friendly settings and services

Several documents mention establishing a non-stigmatizing setting [37, 43] with a youth-friendly environment [13, 31, 37, 38, 40, 43, 52, 70], for example with art [31, 70], couches, music, or recreational activities [38]. Youth-friendly services include not only the environment, but also welcoming and friendly staff [31, 38, 42, 60]. headspace and Jigsaw engaged youth in the development of service settings in order to create a youth-friendly environment [31, 36, 74].

#### Evidence-informed approaches

All programs were found to stress the utilization of evidence-based practices [14, 31, 36, 39, 42, 52, 61, 65, 78], using CBT [14, 52, 65, 78], DBT [52], or other evidence-based or -informed practices [36, 42, 52, 61]. However, details regarding establishing and monitoring fidelity were rarely provided.

#### Partnerships and collaborations

Another core value was forming partnerships and collaborations with a diverse set of agencies. Most programs had partnerships with various stakeholders including youth, families, service providers, community agencies, researchers, hospitals, policy makers, etc. [13, 14, 31, 41, 43, 52]. Partnerships help provide multidisciplinary, co-located services [38] including social, educational, employment services [14], housing support, income assistance [43] and other specialist services [78]. A social network analysis of the headspace staff reported interprofessional collaboration existed in both routine and uncertain situations [81]. In addition, a study conducted with internal and external staff of Youth One Stop Shops reported that individual staff’s trusting personal relationships was crucial for successfully overcoming agency differences, and collaborative care was found to be essential to the successful care of youth with high needs [82].

### Outcome research and program evaluation

Overall, research on youth MH or functional outcomes following intervention was limited, with only 11 documents reporting outcomes (Table 4). One additional article reported youth outcomes; however, the focus was on evaluating an intensive mobile outreach subcomponent rather than overall integrated care [83]. Outcomes reported were predominantly short-term, with long-term follow-up data lacking. Only one evaluation (non-RCT) included a comparison group [84], and few studies provided comparative outcome data to contextualize the magnitude of reported outcomes (exceptions: [51, 73]). Methods of measuring outcomes varied; however, several studies reported using standardized self-report and clinician-rated measures [45, 51, 73, 83–86]. Three documents provided some outcomes information, but did not specify the measures used [37, 38, 87]. In addition to the 11 documents with youth outcome information, three articles reported on case study findings; of them, one utilized a standardized self-report measure [65], whereas two did not [61, 78]. Seven documents discussed future outcome measurement plans, spanning a total of three ICYSH models, all in Canada [13, 39, 41, 43, 44, 52, 88]. Generally, positive outcomes, particularly improvements in psychological distress and psychosocial functioning have been found [37, 45, 73, 84]. Additional evaluation projects are under way by ACCESS Open Minds using a stepped-wedge design [39, 40] and YouthCan IMPACT using a pragmatic randomized controlled trial [52]. ACCESS Open Minds and the Foundry also plan to test the effectiveness of the individual interventions being used [13, 43], adding to the intervention evidence base.

**Table 4:** Youth outcome research within integrated community-based youth service hub models

Program	Sample size (N)	Outcome measures	Assessment time points	Comparative data	Key results
headspace [51]	890	K10 SOFAS	Initial assessment 6th session 10th session	Different staging groups	Attenuated syndrome (stage 1b) youth used significantly more services than help-seeking (stage 1a) youth, including significantly higher rates of psychotropic medication prescription (9.3% vs. 43.6%) At service entry, Stage 1a youth started with significantly lower levels of psychological distress and significantly higher levels of functioning and showed improvement only in psychological distress over 10 sessions Stage 1b youth remained impaired on both measures after 10 sessions but showed modest improvements in levels of psychological distress and functioning
headspace [84]	26,058 headspace clients	K10 SOFAS Surveys	Varied—last recorded K10	Normative population data Other treatment group No treatment group	Results indicate a small program effect Psychological distress of almost half of headspace clients decreased, with 13.3% experiencing a clinically significant reduction, 9.4% a reliable reduction, and 24.3% an insignificant reduction Psychological distress did not change for almost 29%, and increased for nearly 1 in 4. Youth with only 2–3 service occasions were overrepresented in these groups Suicidal ideation reduced significantly even among youth who showed insignificant or no reduction in psychological distress Youth with improved mental health showed positive economic and social outcomes Reduction in psychological distress over time for headspace group was significantly greater than the other treatment and no treatment groups
headspace [74]	70 youth 20 carers	Surveys Semi-structured interviews	Baseline Varied—Wave 1 time point	None at this Wave	92% reported improved mental health since coming to headspace 79% of youth 12–17 and 48% of youth 18–25 reported improved education engagement 71% of youth 14–17 and 55% of youth 18–25 reported improved work ability Youth reported improved relationships with family (12–17: 81%; 18–25: 58%) and friends (12–17: 72%; 18–25: 58%) 54% reported improved physical health Frequency of using at least one illicit substance decreased from 63 to 40%; three-quarters reported better managing emotions without substance use 85% of carers very satisfied with outcomes from child’s headspace involvement
headspace [73]	24,034 headspace clients; 651 at 90-day follow up	K10 SOFAS	K10: prior to 1st, 3rd, 6th, 10th, and 15th visits SOFAS: each visit 90 days after ending services	Comparative outpatient data for RCI score Netherlands mental health clinic NOCC report	Psychological distress significantly reduced in more than one-third of youth Psychosocial functioning improved for a similar proportion 60% experienced significant change when considering improvement in either measure Improvements for youth with greater distress and poorer functioning at intake seen among those who attended more sessions Rate of reliable improvement in psychosocial functioning higher than Netherlands mental health clinic serving similar age range (31 vs. 19%) Outcomes similar to child/adolescent results of NOCC report; higher than adult findings
Jigsaw [45]	709 (first session) 315 (final session)	CORE questionnaire (12–16 year olds: YP-CORE; 17–25 year olds: CORE-10)	First session Final session (average is 4.4 sessions over 13 weeks)	None	Significant differences in pre- and post-intervention levels on both measures 89% showed clinical levels of psychological distress pre-intervention, with 52% reporting moderate/severe or severe levels of psychological distress At final session, majority had healthy (47.2%) or low (28.8%) levels of psychological distress 62% showed reliable and clinically significant improvement on the CORE-10; 22% showed reliable improvement only 68% showed reliable improvement on the YP-CORE
Mom Power group (Corner Health Center) [87]	23	Primarily self- rating scales Not further specified	Post-intervention (10 weeks)	None	Improvements in depression and post-traumatic stress disorder symptoms and decreased rates of psychiatric diagnoses post-intervention Self-rate as less guilt and shame regarding parenting skills post-intervention
Spilstead Model [85]	42	PSI Being a parent scale CBCL Brigance Screen NCFAS Language Assessments GAS Speech measures	Prior to service entry 12 months post admission	None	Large effect size changes in child-focused outcomes including externalizing behaviors, child well-being, and parent–child interactions 71% of children with delays in the clinical range upon initial developmental screening were within the normal range post-treatment; 41% moved from the below average range to scores within the normal range in language development
Youth One Stop Shops [38]	Not specified	Not specified	Not specified	Not specified	94% of clients surveyed believed services effective in improving health and well-being Little robust evidence of health outcomes for youth
Youth One Stop Shops [127]	Total N = 333 Short-term: N = 272; long-term: N = 257	Rating scale	July–August 2012 July–Dec 2012 1st visit—Dec 2012	None	Short term—10% deteriorated, 56% unchanged, 34% improved Long term—17% deteriorated, 37% unchanged, 46% improved Youth with complex needs—3% deteriorated, 39% unchanged, 58% improved 90% of youth and 80% of caregivers interviewed reported the integrated services were a crucial contributor to change
Youthspace; now Forward Thinking Birmingham [37]	Not specified	Not specified	Not specified	Not specified	Only 10% of those referred to the service required secondary care Majority of youth responded well to one-off expert assessment and personalized plan, brief to medium psychological intervention and other support networks 32% were signposted to support network for focused work relating to education, employment and training, with 65% having positive outcome
Youth Wellness Centre [86]	Ranges: 17–44	K10 DERS BSL-23 GAIN	Intake 90 days post-intake	None	Significant reduction in psychological distress (K10), emotional dysregulation (DERS), and borderline symptoms (BSL-23) at 90 days post-intake Significant reduction in days bothered by mental health problems, days not meeting responsibilities, and days with problems paying attention as measured by GAIN Significant increase in family relationships satisfaction, general happiness (GAIN)

*K10* Kessler-10, *SOFAS* Social and Occupational Functioning Assessment Scale, *CORE* Clinical Outcomes in Routine Evaluation, *YP-CORE* Young Person’s Clinical Outcomes in Routine Evaluation, *PSI* Parenting Stress Index, *CBCL* Child Behavior Checklist, *NCFAS* North Carolina Family Assessment Scale, *GAS* Goal Attainment Scaling, *DERS* Difficulties in Emotion Regulation Scale, *BSL-23* Borderline Symptom List-23, *GAIN* Global Appraisal of Individual Needs, *RCI* Reliable Change Index, *NOCC* National Outcomes and Casemix Collection

## Discussion

This scoping review identified international ICYSH models for addressing youth MH needs. Common principles and characteristics of these models are summarized. This review provides a synthesis of the components of such models and can serve as a foundation for informing replication, evaluation and implementation of ICYSHs, which have been rapidly proliferating [13, 14]. However, limited information was provided in many domains, raising questions about the availability of sufficient detail to achieve these goals.

ICYSH models showed some similar characteristics, providing a starting point for replication. Many have focused on early intervention for adolescents and young adults [14, 34, 36, 39, 43, 52, 78], and on addressing the unmet need for services across the transition to adulthood [12]. Nearly all models reported trying to develop non-stigmatizing, youth-friendly environments [89]. Reported efforts were consistent with recommended approaches for improving accessibility and youth-friendliness laid out within calls for youth mental health services reform [90]. Little information was available on service providers’ training or specific roles. More information was provided on broad services categories, with mental health, drug and alcohol services, primary care, vocational or other social services, and peer support featured in several models. Evidence-based and brief, solution-focused interventions were promoted, but specific interventions, extent of their use and achievement of fidelity were rarely reported. Some information was identified on care coordination and infrastructure, such as administrative processes, information sharing, and outcome tracking, although many documents did not discuss these aspects. Overall, the variability in the level of detail provided for several of the characteristics examined precludes drawing strong conclusions regarding the extent of differences across models, and does not allow for clear direction regarding replication, evaluation of these components, nor wide scale implementation. Future reporting should follow TIDieR guidelines in order to facilitate replication, evaluation and implementation [91].

However, there was clearer consistency in the aims of ICYSH models. Improved access to care and earlier intervention were frequently highlighted and served as catalysts for model development. Engaging youth and families in service delivery and design were universally evident. Youth-friendly services and environments were emphasized within all models. Disseminating evidence-informed interventions to more youth in need and implementing evidence-based care were also consistently identified. Finally, leveraging partnerships and collaborations to provide multidisciplinary, integrated services or community capacity building was common. Details regarding the ‘how’ of effectively achieving these aims, however, were limited, thus falling short of providing the information required for replication, evaluation and implementation-related decision-making. For example, the literature is lacking sufficient information on the formation and maintenance of partnerships and related agreements, decision-making processes, and fidelity to evidence-based interventions. Moreover, although integration was frequently mentioned as an aim, there was little explanation of how services are integrated on a structural level beyond the role of care coordinators, and measurement of service integration was lacking.

Notably, the review also revealed potential limitations of these models of care. Specifically, limited service availability [33] and workforce shortages [92] are challenges for the field more broadly and also impact ICYSHs. Additionally, several studies suggest that at least some of the youth presenting for services were experiencing more distress and impairment than models may have been primarily designed to address [49, 51, 59, 93], which is a key finding that should be taken into account in the design and development of robust ICYSH models that can meet the needs of all presenting youth. The complexity of managing numerous objectives and components within a comprehensive ICYSH may limit the ability to fully address each aspect simultaneously.

This review also identified a number of other limitations of the literature. Notably, rigorous evidence of improved youth outcomes attributable to these models of care is scarce. The limited outcome research identified was short-term and lacked control groups (see [19] for a thorough review). All models are from high-income countries, limiting generalizability. Also important to note is the limited translating of work into publications; for some models, the bulk of information was derived from grey literature sources. Finally, numerous relevant documents were not captured using integration-focused search terms (see Appendix), raising questions about terminology consistency.

### Implications for research

This review highlights the pressing need for more rigorous research examining youth outcomes within ICYSHs. Notably, robust designs are needed to evaluate both the effect of ICYSHs on youth outcomes and cost-effectiveness compared to usual treatment. Such efforts are underway for the YouthCan IMPACT program, which is comparing ICYSHs to hospital-based adolescent psychiatric services [52]. ACCESS Open Minds includes a cluster RCT design to evaluate implementation processes, but not youth health outcomes [41]. Additional comparative effectiveness trials comparing treatment outcomes within different ICYSH models, utilizing different control interventions, would strengthen the literature (see also [19]). Given that much information was derived from grey literature, findings also suggest the need to translate more of this work into peer-reviewed publications to facilitate replication and rigorous evaluation. Publications regarding ICYSHs need to include greater detail regarding key attributes, especially those considered unique to the model, such as integration of care, partnership agreements, integrated decision-making processes and youth friendliness to allow for replicability [94]. In addition, supplementary material could be provided on project websites to more thoroughly describe models in a level of detail often not possible within the framework of academic publications. Given the lack of detail on certain aspects of these models, there is insufficient information currently available in these areas to guide implementation.

Utilizing common language and terminology regarding service integration would also improve information sharing and replicability. Reaching a consensus on the most essential elements of these models should be prioritized, for example the types of early interventions that should to be included, but also other characteristics such as types of service providers and other site features. Findings from this review will inform the appraisal of key features of such models, as does the recent review by Hetrick et al. [19]. Additionally, research within established programs could address challenges related to delivering and tracking the use of evidence-based practices in community settings. Finally, using common, core outcome measures [95] to track outcomes and other aspects of ICYSHs would help facilitate collaborative research efforts, support future meta-analyses, and more rapidly advance the field.

### Implications for practice

The finding that youth are presenting to ICYSHs with high levels of distress and impairment—some with more serious needs than models were designed for—has implications for service availability, accessibility, resource allocation, and training. If ICYSH models become a default “stop gap” for youth with more serious difficulties due to a lack of appropriate services elsewhere, their ability to address the needs of youth with emerging MH concerns may be limited. Having clear processes in place supporting a no-wrong-door approach and linking youth with the most appropriate services could lessen these concerns. Leveraging existing service relationships and creating new linkages may be necessary to ensure that youth receive appropriate, comprehensive, multidisciplinary services. These linkages may cross academic-community divides, which may require new ways of working collaboratively (e.g., medical vs. community mental health model). Finally, defining a core basket of services for ICYSH models, as is being discussed in the broader mental health services field (e.g., [96]), will also be important for clarifying the needs that can effectively be met and ensuring that service providers receive appropriate specialized training.

### Implications for policy

This literature synthesis can guide policymakers interested in transforming the youth mental health service system through ICYSH implementation. Policymakers should be aware of the identified deficits in the mental health service system driving the development of ICYSH models and their potential to address these pressing concerns. Policy planning, communication, and funding complexities should be taken into account since comprehensive, cross-sectoral services may span multiple government departments (e.g., youth services, health services, education). Policymakers should be aware that creating ICYSH models does not necessarily require building new services, but can be accomplished by integrating existing services and leveraging existing partnerships, which is encouraging for financial feasibility.

Although much remains to be determined, particularly with regard to outcomes, the profiles of youth best served by such models, cost-effectiveness, and optimal components, this review provides a starting point for understanding the common characteristics of ICYSH models that are likely to be important targets for replication, evaluation, service development efforts and funding. Research granting agencies are encouraged to support rigorous evaluations of ICYSH models to further inform development. Results from economic analyses (e.g., [41, 52]) will have particularly important implications for policy decisions. Policymakers are encouraged to continue to monitor this burgeoning field for enhanced guidance as they develop and expand ICYSHs internationally.

## Limitations

There are limitations of this review that warrant consideration. Although efforts were made to ensure that the review is as thorough and comprehensive as possible, there is inevitably unidentified relevant literature. In particular, the grey literature search was representative, not exhaustive. Additionally, this review was limited to English publications. Documents were found to originate from high-income countries. This may affect the generalizability of the findings to low- or middle-income countries. Information was generally not available on funding models supporting the various ICYSH initiatives; this should be further explored to support new implementation initiatives. To allow for in-depth examination of ICYSHs, this review did not include literature on integrating behavioral health services into existing primary care or school settings, although there may be relevant findings from these areas. Aspects of primary care or school-based models should also be considered in future research to determine the ideal setting. Finally, in accordance with scoping review methodology [21], the quality of studies included in this review was not assessed.

## Conclusions

This review brings together literature on ICYSH models for youth MH from diverse sources and examines their common principles and characteristics. ICYSHs are poised to address many of the most pressing youth mental health service concerns by uniting traditionally fragmented services into single, youth-friendly settings, improving early access to evidence-informed interventions, engaging youth and families, and drawing on the strengths of cross-disciplinary and multi-sectorial collaborations. Future efforts to provide comprehensive descriptions and replicate these models, evaluate youth outcomes and identify the most critical components and processes for ICYSHs will further strengthen these models of care.

## Data Availability

The search strategy is available as an Appendix.

## Appendix: Search strategy: Medline-OVID

1. Delivery of Health Care, Integrated/ (10004)
2. (integrat* adj2 (care or treatment* or therapy or team* or services)).kf,ti.
3. (integrat* adj2 (care or treatment* or therapy or team* or services)).ab. /freq = 2
4. (shared adj2 (care or treatment* or therapy or team* or services)).ti,kf.
5. (multidisciplinary adj2 (care or treatment* or therapy or team* or services)).ti,kf.
6. (multidisciplinary adj2 (care or treatment* or therapy or team* or services)).ab. /freq = 2
7. (shared adj2 (care or treatment* or therapy or team* or services)).ab. /freq=2
8. (shared adj2 (care or treatment* or therapy or team* or services)).ti,kf.
9. (multi-disciplinary adj2 (care or treatment* or therapy or team* or services)).ti,kf.
10. (multi-disciplinary adj2 (care or treatment* or therapy or team* or services)).ab. /freq = 2
11. (coordinat* adj2 (care or treatment* or therapy or team* or services)).ti,kf.
12. (coordinat* adj2 (care or treatment* or therapy or team* or services)).ab. /freq = 2
13. (co-ordinat* adj2 (care or treatment* or therapy or team* or services)).ti,kf.
14. (co-ordinat* adj2 (care or treatment* or therapy or team* or services)).ab. /freq = 2
15. (multiagency adj2 (care or treatment* or therapy or team* or services)).ti,kf.
16. (multiagency adj2 (care or treatment* or therapy or team* or services)).ab. /freq = 2
17. (joint working adj2 (care or treatment* or therapy or team* or services)).ti,kf.
18. (joint working adj2 (care or treatment* or therapy or team* or services)).ab. /freq = 2
19. one stop shop.tw.
20. system of care.ti,kf.
21. system of care.ab. /freq = 2
22. 1 or 2 or 3 or 4 or 5 or 6 or 7 or 8 or 9 or 10 or 11 or 12 or 13 or 14 or 15 or 16 or 17 or 18 or 19 or 20 or 21
23. Mental Health/
24. mental health.ti,kf.
25. exp Mental Health Services/
26. exp mental disorders/
27. ((behavio?r* or mental) adj2 (health or disorder* or problem*)).ti,kf.
28. psychiatry/ or adolescent psychiatry/ or child psychiatry/ or psychology, child/ or psychology, developmental/
29. ((obsessive compulsive or panic or post traumatic stress or eating or bipolar or oppositional defiant or conduct) adj2 disorder*).ti,kf,kw.
30. (depress* or anxiety or substance abuse or drug abuse or alcoholism or alcohol abuse or disruptive behavio?r disorder* or attention deficit or autism or asperger*).ti,kf,kw.
31. (agoraphobia or PTSD or trauma or OCD or anorexia or bulimia or psychosis).ti,kf,kw.
32. or/23–31
33. 22 and 32
34. (pediatric* or paediatric* or child* or adolescent? or youth? or teenag* or teen? or boys or girls or young adult? or emerging adult?).ti,kf,ab,jn.
35. 33 and 34
36. limit 33 to (“all child (0 to 18 years)” or “child (6 to 12 years)” or “adolescent (13 to 18 years)” or “young adult (19 to 24 years)”)
37. 35 or 36
38. limit 37 to english language
39. limit 38 to yr=“2001 -Current”
40. limit 39 to (comment or editorial or letter)
41. 39 not 40
